# MicroRNAs, miR-23a-3p and miR-151-3p, Are Regulated in Dentate Gyrus Neuropil following Induction of Long-Term Potentiation *In Vivo*

**DOI:** 10.1371/journal.pone.0170407

**Published:** 2017-01-26

**Authors:** Brigid Ryan, Barbara J. Logan, Wickliffe C. Abraham, Joanna M. Williams

**Affiliations:** 1 Department of Anatomy, University of Otago, Dunedin, New Zealand; 2 The Brain Health Research Centre, University of Otago, Dunedin, New Zealand; 3 Brain Research New Zealand, Rangahau Roro Aotearoa, University of Otago, Dunedin, New Zealand; 4 Department of Psychology, University of Otago, Dunedin, New Zealand; Nathan S Kline Institute, UNITED STATES

## Abstract

Translation of synaptic mRNA contributes to alterations in the proteome necessary to consolidate long-term potentiation (LTP), a model of memory processes. Yet, how this process is controlled is not fully resolved. MicroRNAs are non-coding RNAs that negatively regulate gene expression by suppressing translation or promoting mRNA degradation. As specific microRNAs are synaptically located, we hypothesized that they are ideally suited to couple synaptic activation, translational regulation, and LTP persistence. The aim of this study was to identify LTP-regulated microRNAs at or near synapses. Accordingly, LTP was induced unilaterally at perforant path-dentate gyrus synapses in awake adult Sprague-Dawley rats. Five hours later, dentate gyrus middle molecular layer neuropil, containing potentiated synapses, was laser-microdissected. MicroRNA expression profiling, using TaqMan Low Density MicroRNA Microarrays (n = 4), identified eight regulated microRNAs. Subsequent individual TaqMan assays confirmed upregulation of miR-23a-3p (1.30 ± 0.10; p = 0.015) and miR-151-3p (1.17 ± 0.19; p = 0.045) in a second cohort (n = 7). Interestingly, bioinformatic analysis indicated that miR-151-3p and miR-23a-3p regulate synaptic reorganisation and transcription, respectively. In summary, we have demonstrated for the first time that microRNAs are regulated in isolated neuropil following LTP induction *in vivo*, supporting the hypothesis that synaptic, LTP-responsive microRNAs contribute to LTP persistence via regulation of the synaptic proteome.

## Introduction

Long-term potentiation (LTP) is a form of synaptic plasticity whereby high-frequency stimulation (HFS) induces a long-lasting enhancement of synaptic transmission. LTP is widely accepted as an excellent model for the molecular mechanisms that mediate long-term information storage in the brain because many of its key properties are analogous to those of long-term memory, including input specificity, rapid induction, cooperativity, and associative interactions [1]. Furthermore, compelling evidence supports the involvement of LTP-type plasticity in memory [2]. While the cellular mechanisms underlying LTP induction have been thoroughly characterised [3], the detailed mechanisms of LTP persistence are not fully understood. Late LTP (L-LTP) is, however, known to be dependent on protein synthesis [1], from both extant dendritic mRNA [4] and newly transcribed mRNA [5]. Indeed, our recent genome-wide expression profiling studies showed that the LTP-related transcriptional response extends much further than the immediate period following LTP expression: mRNAs are differentially expressed up to at least 24 h later [6, 7]. These LTP-regulated gene networks appear to be subject to a variety of regulatory systems, including post-transcriptional control by microRNAs (miRNAs) [6]. MiRNAs were predicted to function as hub molecules in LTP-related gene networks 5 h and 24 h after LTP induction and we and others have shown that the levels of miRNA are altered in response to plasticity [6, 8–13].

Synaptic miRNA expression is of particular interest [14]. MiRNAs and components of miRNA processing machinery are present at mammalian synapses [15] and synaptic activity has been shown to trigger local miRNA processing, suggesting a possible mechanism for expression of mature miRNAs specifically at potentiated synapses [16]. Further, translation of mRNA in biochemically isolated synapses has been shown to be regulated by miR-134-5p [17].

We hypothesize that miRNA levels are altered at synapses in response to LTP induction, and that these miRNAs may be ideally suited to couple synaptic activation, translational regulation, and LTP persistence. To date, however, the subcellular localisation of miRNA expression has received little attention. Investigations into miRNA expression after LTP induction have quantified miRNA levels in whole dentate gyri [6, 8, 10, 12, 13], whole hippocampal slices [9, 11], or the whole CA1 region of hippocampal slices [18]. The only more detailed analysis comes from one report that miR-132-3p upregulation is restricted to granule cell somata 2 h after LTP induction *in vivo* [13].

The aim of this study was to profile miRNA expression in the dentate gyrus middle molecular layer (MML) neuropil after LTP induction at perforant path-dentate gyrus synapses *in vivo*.

## Materials and Methods

### Induction of long-term potentiation in freely moving animals

Adult male Sprague-Dawley rats were implanted with stimulating and recording electrodes in the hippocampus under ketamine (75 mg/kg) and Domitor (0.5 mg/kg) anaesthesia, as reported [6]. We used an established LTP induction paradigm (50 trains of brief 400 Hz delta-burst stimulation) known to induce the most persistent form of LTP, LTP3 [19]. The implanted but untetanised hemisphere served as a within-animal control. Animals were randomly allocated to receive tetanisation to the left (n = 8) or right (n = 4) hemisphere after a 20–30 min period of baseline test pulses administered bilaterally (0.05–0.017 Hz, 150 μs half-wave duration). Following tetanisation, bilateral test pulses were resumed. Responses were recorded for 60 min after the last train, and then again for 30 min beginning 4.5 h after the last train. The averages of the last 20 responses from both recording periods were calculated and expressed relative to the average baseline response. The criteria for LTP were ≥ 10% increase in field excitatory post-synaptic potential (fEPSP) slope and ≥ 100% increase in population spike (PS) amplitude, persisting for 5 h post-tetanisation. Immediately following the second recording period, animals were deeply anaesthetised with halothane and decapitated. Protocols were approved by the University of Otago Animal Ethics Committee.

### Isolation of dentate gyrus subregions

Immediately following decapitation, whole brains were flash-frozen in dry ice-cooled isopentane at ^-^90°C for 4 min. Coronal sections of the dorsal hippocampus (20–35 μm) were cut (Leica CM1950 cryostat; Leica Microsystems) between approximately 2.9 mm and 4.1 mm posterior to bregma and thaw-mounted onto UV-treated polyethylene naphthalate slides. Cryosections were post-fixed (75% EtOH), stained (0.05% thionin), and dried for 15 min at 40°C. The dentate gyrus MML and granule cell layer (GCL) were laser microdissected (Leica LMD6500), collected in Lysis Buffer (Norgen Biotek Total RNA Extraction Kit) with added β-mercaptoethanol (0.1%), and vortexed for 5 s. Laser microdissection ablated the tissue around the margin of the cut, so an area just larger than the MML by the width of the laser cut was dissected to account for this. Despite careful cutting, there may have been inclusion of small amounts of outer molecular layer and/or inner molecular layer in the MML sample.

### RNA purification

The total volume of each sample was adjusted to 600 μL in Lysis Buffer (Norgen Biotek) and total RNA was isolated from matched control and tetanised MML and GCL tissue using the Total RNA Purification Kit (Norgen Biotek), including an on-column DNase treatment. RNA quality and quantity were determined using the NanoDrop^™^ 1000 Spectrophotometer (Thermo Scientific).

### TaqMan Low Density MicroRNA Microarrays

TaqMan Low Density microRNA Arrays (TLDAs) were used to quantify the expression of miRNAs in the MML in response to tetanisation. MiRNA expression in the MML was quantified 5 h after LTP induction, because previous research has indicated that miRNAs function as hubs in LTP-related gene networks at this time-point (Ryan et al., 2012). TLDAs were run using MegaPlex^™^ Pools with pre-amplification and Rat Card A according to the manufacturer’s instructions. Each array contained one reaction for each miRNA; i.e., there were no technical replicates. One animal was excluded from this analysis after preliminary investigation indicated technical inconsistencies in the tetanisation sample.

### Single-plex RT-qPCR

Individual RT-qPCR experiments were conducted according to MIQE (minimum information for publication of quantitative real-time PCR experiments) guidelines [20]. Total RNA (10 ng per 15 μL reaction) was converted to cDNA using the TaqMan^®^ MicroRNA Reverse Transcription Kit (Applied Biosystems). TaqMan^®^ MicroRNA Assays were performed on the Applied Biosystems 7500 Real-Time PCR System according to manufacturer’s instructions, except that RNA input was increased to 4 ng/μL from the recommended 2 ng/μL and RT product was left undiluted, instead of the recommended 1:15 dilution, in cases where preliminary standard curve data indicated that cycle quantification (Cq) values for the lower concentration were close to the limit of accurate quantification (> 30). Reaction efficiency was determined experimentally by performing qPCRs using serial dilutions of cDNA (standard curve method).

### Statistical analysis

Before determining differential expression using TLDAs, Cq values were filtered to remove values above 35 and any datasets with missing values (due to Cq >35 or undetermined). A range of different normalisation strategies were compared to determine which was optimal for this dataset: geometric mean, the small non-coding Y RNA (Y1) endogenous control, U6 small nuclear RNA (U6) endogenous control, miR-16 endogenous control, invariant endogenous controls, scale rank invariant, norm rank invariant, and quantile. Invariant endogenous controls were chosen using the NormFinder algorithm [21]. Normalisation strategies that reduced the mean variance in miRNA expression between samples were considered to be superior, because this is indicative of the removal of technical variation in the data [22]. Variance was determined using standard deviation (SD). Normalisation strategies were applied using the R package ‘HTqPCR’. The endogenous control, miR-301b, was selected as the optimal normalisation strategy for MML data.

We used dual criteria to determine differential expression between the tetanised and control hemispheres: fold change (FC) ± 15% and p < 0.05 (one-sample t-test with a theoretical mean of 1). FC was calculated using the transformation 2^-ΔΔCq^. Two-tailed t-tests were used to analyse the TLDA data; one-tailed t-tests were used to validate the hypotheses generated from these analyses. We used unadjusted p-values to analyse the TLDA data in order to decrease the likelihood of false negatives. Although this approach was likely to inflate the number of false positives, this was mitigated by validating all broad screen results using RT-qPCR in a separate group of animals. Outliers were identified using Grubb’s test. GCL data were normalised to U6. Normal distribution of the data was confirmed using the D’Agostino and Pearson omnibus normality test (p > 0.05). All data are presented as mean ± SEM.

### Bioinformatic analysis

In order to investigate the function of regulated miRNAs, we used nine target prediction algorithms: Probability of Interaction by Target Accessibility (PITA) v6, DIANA-microT v3.0, doRiNA (PicTar2), miRDB v4, miRWalk (March 2011), rna22 (May 2008), RNAHybrid v2.1, miRanda (August 2010), and TargetScanS v6.2. We used the least stringent algorithm parameters to ensure inclusion of all potential targets. Working on the principle that if a target is consistently predicted by multiple algorithms that use different selection criteria it is more likely that the target prediction will be accurate, we only included targets that were predicted by at least four algorithms. Putative target gene lists were cross-checked with a list of mRNAs that are expressed in rat hippocampal neuropil [23] to ensure co-expression with the regulated miRNA.

The function of predicted targets was analysed using the Functional Annotation Clustering Tool in DAVID (Database for Annotation, Visualization and Integrated Discovery) [24, 25]. The Functional Annotation Clustering tool uses a modified Fisher Exact test to determine the probability that a group of genes is involved in a particular biological function, and then groups related biological functions together in a ‘cluster’. Each cluster is given an ‘Enrichment Score’, which is the negative log of the geometric mean of all of the p-values in a given cluster. An Enrichment Score > 1.3 was considered statistically significant (equivalent to p < 0.05). DAVID analyses were performed using medium stringency settings, with all mRNA expressed in rat hippocampal neuropil (2931 transcripts) [23] as the background.

## Results

### Induction of robust LTP in the perforant path of freely moving adult rats

**Fig 1:**
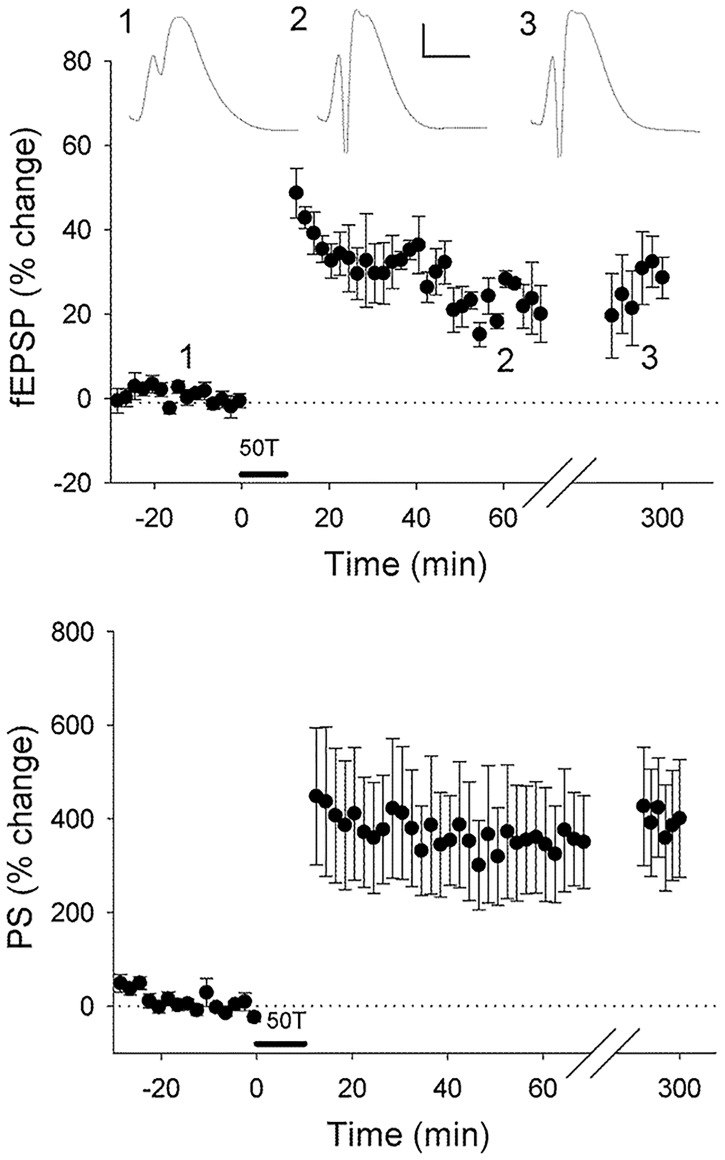
Induction of robust LTP at perforant path-granule cell synapses in awake adult rats. Average (± SEM) field excitatory postsynaptic potential (fEPSP) and population spike (PS) responses expressed as percentage of baseline values. Inset waveforms are averages of 10 sweeps taken just before tetanisation (1), 15–20 min after (2), and 5 h after (3). Calibration bars: 5 ms, 5 mV. 50T: tetanisation paradigm consisting of 50 trains of 400 Hz stimulation.

As we have shown previously using whole dentate gyrus that specific miRNA levels are altered 5 h post-LTP induction, here we focused on this timepoint. Using our established tetanisation protocol [19], we induced robust LTP at perforant path-dentate gyrus synapses in awake, freely moving animals (20 min post-HFS: fEPSP slope, 33 ± 3%; PS amplitude, 370 ± 68%; 5 h post-HFS: fEPSP, 29 ± 4%; PS, 400 ± 62%, n = 11; Fig 1).

### MiR-23a and miR-151-3p are upregulated 5 h after LTP induction *in vivo*

**Fig 2:**
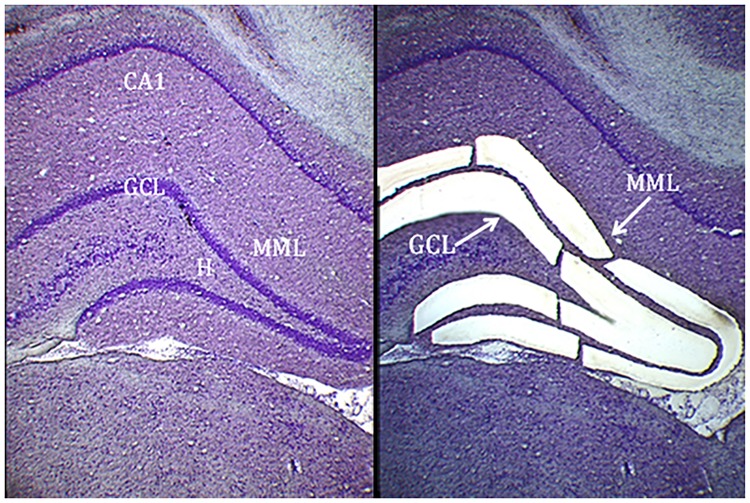
Laser microdissection of the dentate gyrus middle molecular layer from a thionin-stained 35 μm section of rat brain. Intact section (left) and section after microdissection of the middle molecular layer and granule cell layer (right). GCL: granule cell layer; H: hilus; MML: middle molecular layer; CA1: cornu ammonis 1.

To test our hypothesis that miRNAs are regulated in the MML neuropil that includes potentiated synapses 5 h after LTP induction, we carried out miRNA expression profiling of matched LTP-stimulated and control MML tissue using TLDAs (n = 4; Fig 2). The TLDA method was chosen because RNA sequencing was precluded by low total RNA yield (average 449 ± 273 ng). There were no significant differences in RNA quality or quantity between the stimulated and control groups (two-tailed t-tests; average concentration 9.0 ± 1.7 ng/μL; range: 3.7 to 20.8 ng/μL; A260/A280 ratios > 1.7). TLDA analysis showed robust expression of 223 individual miRNAs in the MML (Cq ≤ 35 in all eight samples). It is noteworthy that when these data were compared with our recent RNA-Seq data [26] 32 were found to be expressed exclusively in the MML and 29 were found to be expressed exclusively in the GCL. These data reinforce that we have achieved separation between the GCL and MML.

**Fig 3:**
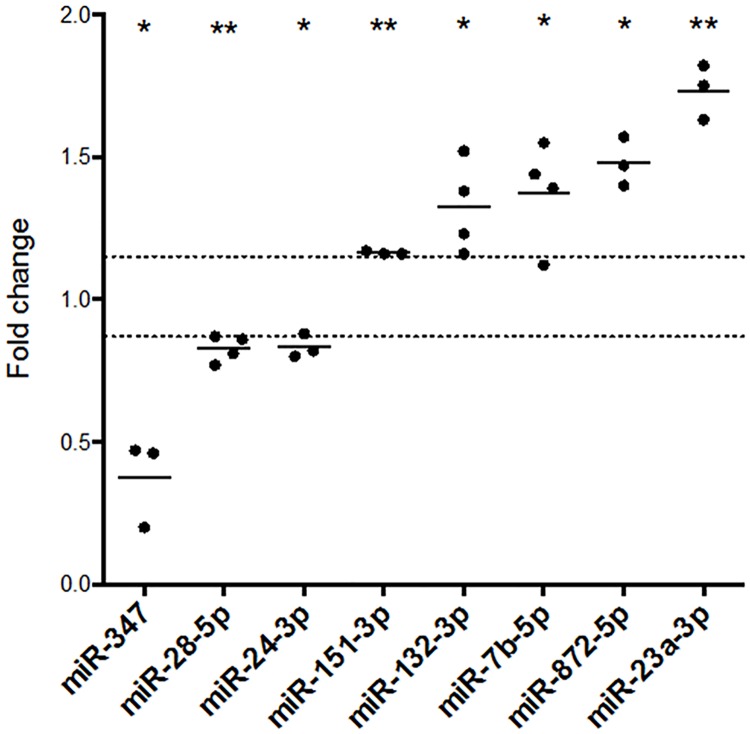
LTP regulates microRNA expression in the dentate gyrus middle molecular layer 5 h post-tetanisation. Regulation of microRNA expression in the middle molecular layer revealed by TaqMan Low Density microRNA Arrays. Eight microRNAs were found to be differentially expressed using dual selection criteria (unadjusted two-tailed Student’s t-test p < 0.05; fold change ± 15%): three were downregulated; five were upregulated. Expression values: individual fold changes and means. Outliers were removed using Grubb’s Test. All data were normalised to miR-301b. Dotted lines indicate cut-off for fold change criterion (± 15%).* p < 0.05; ** p < 0.01; n = 3–4.

Using dual selection criteria (FC ± 15% and p < 0.05), we identified eight miRNAs that were differentially expressed in response to tetanisation (Fig 3). While there was no correlation between LTP magnitude and miRNA fold change (data not shown), five miRNAs were upregulated: miR-132-3p (FC = 1.32 ± 0.08; p = 0.029); miR-7b-5p (FC = 1.38 ± 0.09; p = 0.025); miR-151-3p (FC = 1.16 ± 0.004; p < 0.001); miR-872-5p (FC = 1.48 ± 0.05; p = 0.011); and miR-23a-3p (FC = 1.73 ± 0.05; p = 0.005). Three miRNAs were downregulated: miR-347 (FC = 0.38 ± 0.09; p = 0.019); miR-28-5p (FC = 0.83 ± 0.02; p = 0.005); and miR-24-3p (FC = 0.84 ± 0.02; p = 0.020). The magnitude of the observed changes in miRNA expression, while modest (mean upregulation: 1.41; mean downregulation: 0.68), was consistent with previous findings of LTP-related miRNA expression [6, 8, 12, 13].

**Fig 4:**
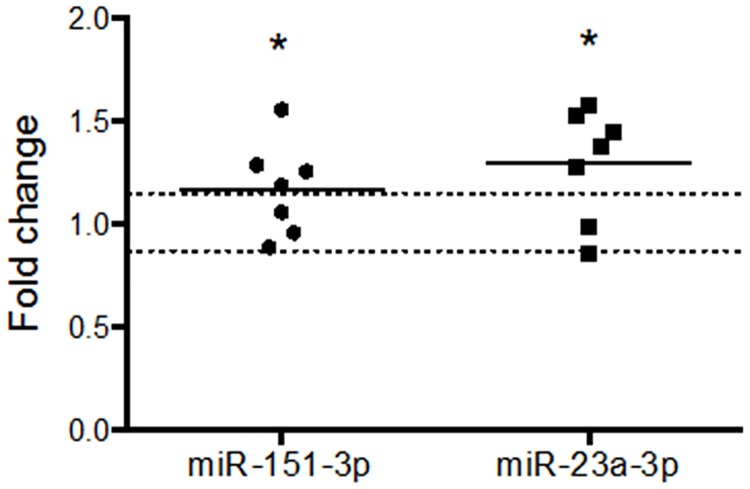
Validation of differential expression of miR-151-3p and miR-23a-3p in the dentate gyrus middle molecular layer 5 h after tetanisation. Upregulation of miR-151-3p and miR-23a-3p was confirmed by single-plex RT-qPCR (dual criteria: one-tailed Student's t-test p < 0.05; fold change ± 0.15). Expression values: individual and mean fold changes. * p < 0.05; n = 7. Dotted lines indicate cut-off for fold change criterion (± 15%). All data were normalised to miR-301b.

Of the miRNAs identified in the discovery phase of our study, two were confirmed as differentially expressed in a second validation study in a separate group of animals (n = 7): miR-23a-3p (FC = 1.30 ± 0.10; p = 0.015) and miR-151-3p (FC = 1.17 ± 0.19; p = 0.045) (Fig 4). These data constitute the first demonstration of miRNA regulation in neuropil in response to LTP induction *in vivo*.

**Fig 5:**
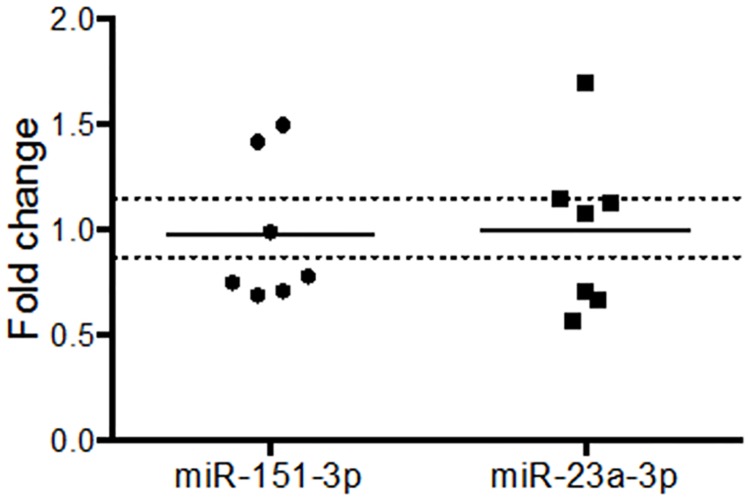
MiR-151-3p and miR-23a-3p are not differentially expressed in the dentate gyrus granule cell layer 5 h after tetanisation. Expression values: individual and mean fold changes determined using single-plex RT-qPCR. Dual criteria for differential expression: one-tailed Student's t-test p < 0.05; fold change ± 0.15. N = 7. Dotted lines indicate cut-off for fold change criterion. Data were normalised to U6.

The magnitude of regulation for miR-151-3p was very similar to that of the TLDA result (FC = 1.17 versus 1.16), whereas regulation of miR-23a-3p was more modest (FC = 1.30 versus 1.73). Interestingly, regulation of both miR-151-3p and miR-23a-3p was specific to the MML. Neither was differentially expressed in the isolated granule cell layer, containing granule cell somata (miR-23a-3p: FC = 1.00 ± 0.15; p = 0.50; miR-151-3p: FC = 0.98 ± 0.13; p = 0.43; n = 7; Fig 5), although miR-132-3p was up-regulated (FC = 1.41; p = 0.03; data not shown) in keeping with previous research [13].

### Predicted function of miR-23a-3p and miR-151-3p

Having found that miR-23a-3p and miR-151-3p were upregulated in the MML 5 h after LTP induction *in vivo*, we next explored the biological significance of this regulation. To this end, we predicted targets of these microRNA and cross-checked these with the set of mRNA known to be expressed in neuropil [23]. Using a consensus approach (i.e. a target must be predicted by at least four algorithms; see Methods and Materials for further detail), our bioinformatic analysis revealed that 356 putative mRNA targets of miR-23a-3p and 74 putative mRNA targets of miR-151-3p are expressed in hippocampal neuropil.

Having identified sets of putative neuropil-resident miR-23a-3p and miR-151-3p targets we probed the function of these mRNA sets using the functional annotation clustering tool in DAVID. The DAVID analysis of the predicted targets of miR-151-3p showed enrichment for the cluster of ontological terms including “Positive regulation of cellular component biogenesis” and “Cell projection organization” (enrichment score = 1.4). This enrichment was due to 9 genes: BBS10, BBS4, CAPZA2, CHL1, DYNC2H1, MAP1B, PFN2, STXBP1, and TSC1.

DAVID analysis showed that the predicted targets of miR-23a-3p were significantly enriched for the cluster of ontological terms, ‘Positive regulation of transcription’ (enrichment score = 1.75). This enrichment was due to 60 genes (S1 Table), including transcription factors (BCLAF1, ABT1, RUNX1T1), zinc finger proteins (CTCF, GZF1, GAZF1, ZC3H8, ZFR, ZBTB38), and the histone acetyltransferase BAZ2B. Intriguingly, BAZ2B is a member of an evolutionarily conserved family of bromodomain proteins which are epigenetic ‘reader’ domain proteins, involved in regulation of non-coding RNA species. Further, we have previously reported that BAZ2B is downregulated in whole dentate gyrus 5 h after LTP induction *in vivo* [6], lending weight to the idea that it is directly regulated by miR-23a-3p.

## Discussion

This study provides the first *in vivo* evidence of miRNA regulation in neuropil following LTP induction of the perforant path in awake rats. Using laser microdissection we were able to profile global miRNA expression at the subcellular level after LTP induction *in vivo*. Through microarray expression profiling and RT-qPCR analysis we have identified two miRNAs in isolated neuropil, miR-23a-3p and miR-151-3p, which are up-regulated 5 h after LTP of perforant path-dentate gyrus synapses *in vivo*.

Regulation was spatially specific in that neither miRNA was regulated in the granule cell layer containing the somata of the postsynaptic cells. Upregulation of miR-23a-3p and miR-151-3p has not been reported in previous studies of miRNA regulation following LTP induction (Wibrand et al., 2010; Ryan et al., 2012b; Wibrand et al., 2012; Joilin et al., 2014; Pai et al., 2014; Park & Tang, 2009; Lee et al., 2012; Gu et al., 2015). As these studies did not quantify miRNAs specifically in the neuropil, it is likely that they were not able to detect such subtle changes in miR-23a-3p and miR-151-3p at the subcellular level.

As both miR-23a-3p and miR-151-3p are known to be expressed in adult rat synapses [27], our findings suggest that these miRNAs may be involved in tight temporal control of protein expression at synapses in response to LTP induction. Further work is needed to test whether the regulation of these miRNAs is dependent on NMDA receptor activation, and to confirm whether these changes in miRNA expression are occurring specifically at potentiated synapses. Alternatively they may participate in synaptic plasticity via depotentiation of neighbouring synapses, thereby increasing the saliency of the potentiated synapses. Investigation of miR-23a-3p and miR-151-3p regulation at different time-points following LTP induction is also warranted. This would provide vital insight into the dynamics of miRNA regulation in response to LTP induction.

The dentate gyrus molecular layer consists mainly of the dendritic trees of neurons that have their somata in the granule cell layer (granule cells, pyramidal basket cells, axo-axonic cells, and hilar commissural-associated pathway-related cells) and is innervated by axons from the entorhinal cortex and other sources, the vast majority of which terminate in the middle and outer molecular layers [28]. It is of note that the molecular layer is not entirely free of neuronal somata as molecular layer perforant path-associated interneurons are also located in the molecular layer. It is also possible therefore, although unlikely, that the observed changes in miR-23a-3p and miR-151-3p expression contribute to plasticity via regulation of the minority synapses on the shafts of inhibitory interneurons or somata of these and other cell populations found in the MML. We also cannot discount the possibility that the observed changes in miR-23a-3p and miR-151-3p are occurring in glia resident in the neuropil; however, there is currently no evidence that either of these miRs is expressed in glia. Localisation of these miRNAs using *in situ* hybridization would be informative, both to confirm expression in the neuropil and to determine their specific cellular origin.

Our observation that upregulation of miR-23a-3p and miR-151-3p is specific to the synaptodendritc compartment, and does not occur in the granule cell somatic layer, suggests that these miRNAs are regulated post-transcriptionally. The compartmentalised, step-wise nature of miRNA biogenesis enables tight spatial and temporal control of miRNA activity. The observed increase in mature miRNA levels could be the result of accelerated precursor processing, or decreased turnover of mature miRNA [29–33].

Bioinformatic analysis of the predicted synaptic targets of miR-151-3p indicated that they contribute to “Positive regulation of cellular component biogenesis” and “Cell projection organization”. This suggests that miR-151-3p regulates synaptic reorganisation, which is associated with L-LTP [34, 35]. The majority of synapses in the mammalian brain are formed on dendritic spines, which exhibit actin-dependent morphological plasticity; moreover, their size has been correlated with synaptic efficacy [36]. Thus, LTP consolidation, and long-term storage of memories, may be achieved by an increase in the size, and therefore strength, at potentiated synapses. LTP-related enlargement of spines is associated with trafficking of polyribosomes from dendrites to spines [37], suggesting a potential link between local dendritic translation and structural synaptic change.

Interestingly, a number of the predicted synaptic targets of miR-151-3p have been linked to intracellular trafficking: profilin 2 (PFN2) [38]; dynein, cytoplasmic 2, heavy chain 1 (DYNC2H1); Bardet-Biedl syndrome 4 (BBS4) [39]; and Bardet-Biedl syndrome 10 (BBS10). The exact function of BBS10 is unknown; however, BBS4 and other BBS family members play a role in retrograde intracellular trafficking, specifically dynein-dependent transport towards the minus-end of microtubules [39]. This suggests that miR-151-3p may regulate receptor/vesicle trafficking at the synapse in response to LTP induction. For example, studies from our laboratory and others have shown that the levels of both AMPA and NMDA receptor subunits are dynamically regulated up to 48 h after LTP induction [40–45].

Bioinformatic analysis suggested that miR-23a-3p is a negative regulator of transcription. Our finding that miR-23a-5p is regulated in the neuropil, and that its predicted target mRNAs are expressed in hippocampal neuropil, raises the intriguing possibility that miR-23a-3p may be involved in communication between activated synapses and the nucleus to regulate LTP-related transcription. Of particular interest is the predicted target BAZ2B, a member of an evolutionarily conserved family of epigenetic ‘reader’ domain proteins that regulate non-coding RNAs. BAZ2B contains a bromodomain that selectively recognises sequences containing acetylated lysine. In addition to its role as a transcriptional regulator, it is possible that BAZ2B functions at the synapse, as lysine acetylation is also common in non-nuclear proteins [46]. For example, lysine acetylation stabilises proteins and protects against degradation via ubiquitination [47]. As we have previously reported that BAZ2B is downregulated in whole dentate gyrus 5 h after LTP induction *in vivo* (Ryan et al., 2012) this predicts that BAZ2B may be directly regulated by miR-23a-3p and contributes to the consolidation of LTP.

## Conclusion

We have demonstrated upregulation of miR-23a-3p and miR-151-3p in the MML 5 h after LTP induction in awake rats, thereby identifying two novel LTP-related miRNAs and demonstrating miRNA regulation in the neuropil in response to LTP induction *in vivo* for the first time. Our previous bioinformatic analysis of LTP-related gene networks predicts that the ongoing and dynamic transcriptional response to LTP induction contributes to alteration of synapses, through regulation of calcium dynamics, protein kinases, glutamate receptors and synaptogenesis [6]. The results of the present study suggest that these changes, which are thought to underlie LTP persistence, may be affected by miRNA activity: the expression of newly-transcribed mRNAs could be fine-tuned by miRNAs specifically at potentiated synapses. There are, however, few reports of a direct effect of altering miRNA activity on LTP maintenance or to related forms of activity-dependent synaptic plasticity such as LTD or depotentiation [14]. Thus further work is required to explore the relationship between miR-23a-3p and miR-151-3p and LTP maintenance.

## Supporting Information

S1 Table Predicted mRNA targets of miR-23a-3p associated with ‘Positive regulation of transcription’. (PDF)
