# Supplementary material for: MicroRNAs, miR-23a-3p and miR-151-3p, Are Regulated in Dentate Gyrus Neuropil following Induction of Long-Term Potentiation In Vivo
Source: PLoS One. 2017 Jan 26;12(1):e0170407. doi: 10.1371/journal.pone.0170407 (PMC5268419; doi:10.1371/journal.pone.0170407)
Supplement: S1 Table — (PDF) [file pone.0170407.s001.pdf]

S1 Table: Predicted mRNA targets of miR-23a-3p

Official Gene Symbol	Gene Name	Official Gene Symbol	Gene Name	Official Gene Symbol	Gene Name	Official Gene Symbol	Gene Name
BCLAF1	BCL2-associated transcription factor 1	ACTN2	actinin alpha 2	HNRPDL	heterogeneous nuclear ribonucleoprotein D-like	PKIA	protein kinase (cAMP-dependent, catalytic) inhibitor alpha
CTCF	CCCTC-binding factor (zinc finger protein)	ABT1	activator of basal transcription 1	IRS2	insulin receptor substrate 2	RXRG	retinoid X receptor gamma
CNOT2	CCR4-NOT transcription complex, subunit 2	ADNP	activity-dependent neuroprotector homeobox	IRF2	interferon regulatory factor 2	RNF10	ring finger protein 10
GPBP1	GC-rich promoter binding protein 1	ATRX	alpha thalassemia/mental retardation syndrome X-linked (RAD54 homolog, S. cerevisiae)	LRPPRC	leucine-rich PPR-motif containing	RUNX1T1	runt-related transcription factor 1; translocated to, 1 (cyclin D-related)
GZF1	GDNF-inducible zinc finger protein 1	AHR	aryl hydrocarbon receptor	KDM4A	lysine (K)-specific demethylase 4A	SAFB	scaffold attachment factor B
JAZF1	JAZF zinc finger 1	ARNT	aryl hydrocarbon receptor nuclear translocator	MED14	mediator complex subunit 14	H3F3B	similar to H3 histone, family 3B; similar to Zgc:56193; similar to H3 histone, family 3A; H3 histone, family 3B; similar to Histone H3.3; similar to histone 1, H2ai
KLF10	Kruppel-like factor 10	BLOC1S2	biogenesis of lysosomal organelles complex-1, subunit 2	MTERF	mitochondrial transcription termination factor	HMGN2	similar to Nonhistone chromosomal protein HMG-17 (High-mobility group nucleosome binding domain 2); high mobility group nucleosomal binding

							domain 2
MEIS2	Meis homeobox 2	BAZ2B	bromodomain adjacent to zinc finger domain, 2B	MAPK14	mitogen activated protein kinase 14	SSBP2	single-stranded DNA binding protein 2
PTK2B	PTK2B protein tyrosine kinase 2 beta	CCK	cholecystokinin	NFIB	nuclear factor I/B	SNCA	synuclein, alpha (non A4 component of amyloid precursor)
SKP1	S-phase kinase-associated protein 1	CBFA2T2	core-binding factor, runt domain, alpha subunit 2, translocated to, 2 (human)	NCOA6	nuclear receptor coactivator 6	TERF2	telomeric repeat binding factor 2
SATB2	SATB homeobox 2	CCND1	cyclin D1	NPM1	nucleophosmin (nucleolar phosphoprotein B23, numatrin)	TRAPPC2	trafficking protein particle complex 2; sedlin-like
SRPK1	SFRS protein kinase 1	CCNL2	cyclin L2	OBFC2A	oligonucleotide/oligosaccharide-binding fold containing 2A	TSNAX	translin-associated factor X
SMAD3	SMAD family member 3	ETV1	ets variant 1	PEBP1	phosphatidylethanolamine binding protein 1	ZC3H8	zinc finger CCCH type containing 8
SMARCC1	SWI/SNF related, matrix associated, actin dependent regulator of chromatin, subfamily c, member 1	FGF2	fibroblast growth factor 2	PBRM1	polybromo 1	ZFR	zinc finger RNA binding protein
ANP32A	acidic (leucine-rich) nuclear phosphoprotein 32 family, member A	FOXK1	forkhead box K1	PDCD4	programmed cell death 4	ZBTB38	zinc finger and BTB domain containing 38
